# From Near-Neutral to Strongly Stratified: Adequately Modelling the Clear-Sky Nocturnal Boundary Layer at Cabauw

**DOI:** 10.1007/s10546-017-0304-8

**Published:** 2017-10-07

**Authors:** P. Baas, B. J. H. van de Wiel, S. J. A. van der Linden, F. C. Bosveld

**Affiliations:** 1 Geoscience and Remote Sensing, Delft University of Technology, PO-Box 5048, Delft, The Netherlands; 2 R&D Observations and Data Technology, Royal Netherlands Meteorological Institute, PO-Box 201, De Bilt, The Netherlands

**Keywords:** Model evaluation, Numerical weather prediction, Single-column model, Stable boundary layer

## Abstract

The performance of an atmospheric single-column model (SCM) is studied systematically for stably-stratified conditions. To this end, 11 years (2005–2015) of daily SCM simulations were compared to observations from the Cabauw observatory, The Netherlands. Each individual clear-sky night was classified in terms of the ambient geostrophic wind speed with a $1\hbox { m} \hbox { s}^{-1}$ bin-width. Nights with overcast conditions were filtered out by selecting only those nights with an average net radiation of less than $-\,30\hbox { W }\hbox {m}^{-2}$. A similar procedure was applied to the observational dataset. A comparison of observed and modelled ensemble-averaged profiles of wind speed and potential temperature and time series of turbulent fluxes showed that the model represents the dynamics of the nocturnal boundary layer (NBL) at Cabauw very well for a broad range of mechanical forcing conditions. No obvious difference in model performance was found between near-neutral and strongly-stratified conditions. Furthermore, observed NBL regime transitions are represented in a natural way. The reference model version performs much better than a model version that applies excessive vertical mixing as is done in several (global) operational models. Model sensitivity runs showed that for weak-wind conditions the inversion strength depends much more on details of the land-atmosphere coupling than on the turbulent mixing. The presented results indicate that in principle the physical parametrizations of large-scale atmospheric models are sufficiently equipped for modelling stably-stratified conditions for a wide range of forcing conditions.

## Introduction

This study investigates to what extent a single-column model (SCM) that is derived from a well-known numerical weather prediction (NWP) model reproduces observed dynamics of the nocturnal boundary layer (NBL) for a wide range of mechanical forcing conditions. To this end, 11 years of daily SCM simulations are compared with observations from the Cabauw observatory in the Netherlands. All individual clear-sky nights are classified in terms of the ambient geostrophic wind speed with a $1\hbox { m }\hbox {s}^{-1}$ bin-width. For each class of geostrophic wind speed, ensemble-averaged profiles and time series of relevant quantities are constructed. As such, this approach provides a detailed picture of the model’s performance for a broad range of stabilities, ranging from near-neutral to strongly-stratified conditions. Such a systematic model evaluation is of particular relevance, since the representation of stably-stratified conditions in NWP models is a longstanding challenge in meteorology (e.g. Viterbo et al. 1999; Brown et al. 2008; Fernando and Weil 2010; Holtslag et al. 2013; Sandu et al. 2013).

While in convective conditions the thermodynamic evolution of the boundary layer may be dominated by turbulence as the major transport mechanism, for stably-stratified conditions processes like radiative transport and land-atmosphere coupling become equally important (Steeneveld et al. 2006; Edwards 2009; Sterk et al. 2013; Pithan et al. 2016). In addition, other processes may add to the complexity of stably-stratified boundary layers such as, for example, momentum transport due to gravity waves (Chimonas and Nappo 1989), low-level jet formation (Banta et al. 2002), weak and non-stationary turbulence (van de Wiel et al. 2002), surface heterogeneity (McCabe and Brown 2007), and the occurrence of fog and dew (Román-Cascón et al. 2016). The various processes and their interactions are often not well understood and/or poorly represented in numerical models. Especially strongly-stratified boundary layers remain a serious challenge for numerical models (Atlaskin and Vihma 2012; Donda et al. 2013).

Sandu et al. (2013) illustrate the complexity of modelling the NBL on a global scale with the European Centre of Medium-Range Weather Forecasting (ECMWF) model. They demonstrate that the parametrization of turbulent transport in stably-stratified conditions affects the representation of the large-scale flow (cf. Beare 2007; Brown et al. 2008). In fact, to optimize model scores on the synoptic scale, many operational models apply much more vertical mixing than can be motivated from observations (so-called ‘enhanced mixing’), although this approach has clear detrimental effects for the representation of the NBL (Brown et al. 2005; Cuxart et al. 2006; Svensson and Holtslag 2009). Furthermore, Sandu et al. (2013) show large impact of the land-atmosphere coupling on the near-surface temperature. Also the representation of (orographically-induced) gravity waves is shown to affect both the NBL representation as well as the large-scale flow. Tsiringakis et al. (2017) hypothesize that small-scale gravity wave drag may explain the discrepancy between observed mixing efficiencies and enhanced mixing that is required by operational NWPs.

The Gewex Atmospheric Boundary Layer Studies (GABLS) intercomparison studies have focused on the complex interactions between different processes in stably-stratified conditions (Cuxart et al. 2006; Svensson et al. 2011; Bosveld et al. 2014). For example, by analyzing results from 19 SCMs, Bosveld et al. (2014) demonstrated that differences in land-atmosphere coupling explained most of the variability in both the near-surface temperature and the longwave incoming radiation among the models. The efficiency of vertical mixing impacted mainly on the boundary-layer height and the wind-speed profiles, but had little consequence for the 2-m temperature.

As demonstrated by, for example, the GABLS intercomparison studies, SCMs are powerful tools with which to study complex interactions between different processes in the atmosphere (cf. Neggers et al. 2012). This is facilitated by the high transparency of the code, the lack of interference with the three-dimensional dynamics, and the low computational costs, which facilitates conducting sensitivity experiments. However, for realistic SCM simulations large-scale forcings are needed, in particular the geostrophic velocity, the vertical velocity, and the advective tendencies of momentum, heat and moisture. For example, omitting advective tendencies leads to strong deviations from reality (Baas et al. 2010; Sterk et al. 2015). But unfortunately, these large-scale forcings are inherently uncertain, which complicates a direct comparison of model results with observations. Baas et al. (2010) demonstrated that considering composite cases has clear advantages over analyzing individual cases. While specific (mesoscale) synoptic disturbances may hamper a one-to-one comparison between observations and model results, in a composite non-systematic perturbations are largely ‘averaged out’.

It is uncertain to what extent NWP models are able to represent the various NBL regimes that have been distinguished in observations (e.g. Mahrt et al. 1998; Grachev et al. 2005; Mauritsen and Svensson 2007; Sun et al. 2012; Acevedo et al. 2015; Mahrt et al. 2015; van Hooijdonk et al. 2015; Monahan et al. 2015). While the precise definitions may vary, most studies define a *weakly stable* regime with strong and continuous turbulence and a *very stable* regime in which turbulence is weak, patchy and/or intermittent. Often, a transitional regime is defined in which the magnitude of turbulent quantities decreases rapidly with increasing stability.

Here, we investigate the performance of the Regional Atmospheric Climate Model (RACMO) SCM (van Meijgaard et al. 2008) for stably-stratified conditions in a systematic way. The aim of the present study is twofold:

- to evaluate the performance of the model against observations for a wide range of stability conditions by means of a classification in terms of the geostrophic wind speed;
- to infer to what extent the model reproduces observed regime transitions in the NBL.

Specific research questions include:

- How does the model respond to changing (mechanical) forcing conditions in comparison with observations?
- Is model performance better for weakly stable conditions than for strongly-stratified conditions?
- How does a turbulence kinetic energy (TKE) scheme with ‘realistic’ mixing characteristics perform compared to a ‘traditional’ enhanced-mixing scheme?
- Does the SCM reproduce characteristics of regime transitions as observed in observational studies?

In related work of the present authors, van der Linden et al. (2017) presented a classification of clear-sky nocturnal boundary layers at Cabauw in terms of the ambient geostrophic wind speed. They concluded that ensemble-averaged variables organize surprisingly well as a function of their corresponding geostrophic wind speed. The current study uses their result as a benchmark for model evaluation. A comparable approach was taken by Bosveld and Beyrich (2004) and Donda et al. (2013), who compared ensemble-averaged wind and temperature profiles from Cabauw with ECMWF model output.

This paper is structured as follows. Section 2 briefly describes the measurements at Cabauw and introduces the SCM. A discussion of relevant model components is included. Section 3 describes the analysis procedure including the classification of nights in terms of the geostrophic wind speed. Section 4 presents the results. First, the classification of model results and observations in terms of geostrophic wind speed is discussed; second, the model’s ability to represent observed NBL regimes is explored; third, the impact of turbulent mixing and the strength of the land-atmosphere coupling on the near-surface inversion are studied with idealized SCM simulations. In Sect. 5 the presented results are discussed in view of findings from earlier studies. Section 6 summarizes the conclusions.

## Observations and Model

### Observations

The Cabauw observatory is located in the western part of The Netherlands (51.971 N, 4.927E) in topographically flat terrain (van Ulden and Wieringa 1996; Monna and Bosveld 2013). The 213-m main tower is surrounded by grassland to at least 200 m in all directions, and at larger distances the terrain is heterogeneous with tree lines and scattered villages. No unambiguous roughness length for momentum, $z_{0\mathrm{m}}$, can be given for the site. The local grassland has $z_{0\mathrm{m}}$ equal to 0.05 m, but the upper levels of the measurement tower experience much higher roughness lengths (Beljaars 1982; Verkaik and Holtslag 2007). Furthermore, $z_{0\mathrm{m}}$ varies strongly with wind direction (Beljaars and Bosveld 1997; Optis et al. 2015).

The physical mechanism through which obstacles extract momentum from the flow (pressure drag) is absent for heat transport. As such, for a model evaluation study a local-scale estimate of the roughness length for heat, $z_{0\mathrm h}$, seems to be appropriate. Bosveld et al. (2014) estimate $z_{0\mathrm h}$ from the observed surface radiation temperature of the local grassland, the air temperature at 1.5-m height, and the sensible heat flux. A typical value of $z_{0\mathrm h} = 0.0015\hbox { m}$ is found.

Here we use data from the period 2005–2015 (11 years). Wind and temperature are measured at 10, 20, 40, 80, 140 and 200 m above the surface with cup-anemometers and shielded Pt500-elements, respectively. In addition, temperature is also measured at 1.5 m height. On the main tower no undisturbed measurements can be made below 20 m. Therefore, observations of 20 m above the surface and lower are taken from a 20-m high auxiliary mast that is located at sufficient distance from the main tower. All components of the radiation flux budget are measured at 1.5 m.

Near-surface turbulent fluxes of heat and momentum are derived from sonic anemometer observations by means of the eddy-correlation technique, where turbulent fluxes are defined *positive* when directed *towards* the surface. As the sonic anemometer is located at only 3 m above the surface, the obtained friction velocity is representative for the smooth grassland directly around the measurement site. It is therefore referred to as the *local* friction velocity. Following Bosveld et al. (2014), we consider this local friction velocity as non-representative for a comparison with numerical model output that is based on a roughness length that is representative for a larger area. Instead, we define a so-called *regional* friction velocity that is derived from the 10-m wind speed and the temperature difference between 10 and 2 m with the profile method. A roughness length of 0.15 m is applied, which is similar to the roughness length taken in the model simulations. Unless specifically mentioned, in this study we use this *regional* friction velocity.

As explained in Sect. 3, we classify selected nights according to the local surface pressure gradient, which is derived from 24 surface pressure observations in a radius of approximately 200 km around Cabauw (cf. Bosveld et al. 2014). As an approximation to the surface pressure field a second-order polynomial is fitted through the observations. In the presentation of the results, the local pressure gradient is converted to a geostrophic wind speed, $U_\mathrm{g}$.

### Model

For the period 2005–2015 daily model simulations have been performed with the RACMO SCM, which is based on Cy31r1 of the Integrated Forecasting System (IFS) of the ECMWF (ECMWF 2007). The main difference between RACMO (van Meijgaard et al. 2008) and the original IFS model is the parametrization of turbulent mixing. Instead of the original first-order closure model a turbulence kinetic energy (TKE or *E*) closure model is used. The individual terms of the TKE equation are parametrized in terms of the local mean gradients of wind, temperature, and TKE. For the computation of the eddy diffusivities of momentum and heat the diagnostic length-scale formulation proposed by Lenderink and Holtslag (2004) is used.

The length-scale formulation consists of two parts. For convective to near-neutral (including weakly stable) conditions the so-called integral length scale applies, which includes the effect of layer-stability. For very stable conditions the integral length scale is overridden by a separate ‘stable’ length scale, $l_\mathrm{s}$, which is given by

1 $$\begin{aligned} l_s =c_{m,h} \frac{\sqrt{E}}{N}, \end{aligned}$$

with $c_m =c_h \left( {1+c_p Ri_g } \right)$ and *N* denotes the Brunt–Väisälä frequency defined as $\sqrt{\frac{g}{\theta }\frac{\mathrm{d}\theta }{\mathrm{d}z}}$ and $Ri_{g}$ the local gradient Richardson number defined as $g/\theta \,\, \mathrm{d}\theta /\mathrm{d}z \,\,/\,\, (\mathrm{d}U/\mathrm{d}z)^{2}$. Wind speed, potential temperature, and the acceleration due to gravity are denoted by *U*, $\theta$, and *g*, respectively. $c_{h}$ and $c_{p}$ are model constants. The integral length scale and the stable length scale are connected by inverse quadratic interpolation.

**Fig. 1:**
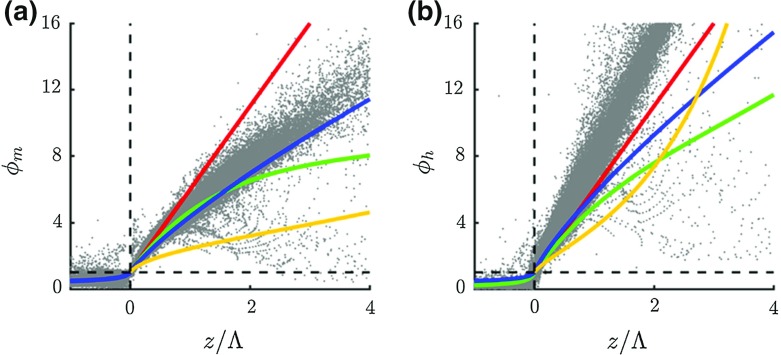
Dimensionless gradients of wind, $\varphi _{m}$, and temperature, $\varphi _{h}$, as a function of the local stability parameter $z/\Lambda$ as diagnosed from 1 year of model output (grey dots). Red lines indicate $1 + 5\, z/\Lambda$ (Dyer 1974), green lines the Beljaars and Holtslag (1991) formulation, blue lines the relations proposed by Duynkerke (1991), and yellow lines the formulation that is used in the ECMWF model

Baas et al. (2008) analyzed the scaling behaviour of the stable length scale, in particular the relation between the dimensionless gradients of momentum and heat versus the stability parameter $z/\Lambda$. Here $\Lambda$ denotes the local Obukhov length. They demonstrated that the values of model constants $c_{h}$ and $c_{p}$ are directly related to the slope of the resulting flux-gradient relations in the stable limit, which enables a more physically-based choice of parameter settings. For the present study we apply $c_{h} = 0.11$ and $c_{p} = 2$. Figure 1 shows the flux-gradient relations for momentum and heat as diagnosed from 1 year of SCM simulations together with several formulations taken from the literature. As a result of a matching procedure between the integral length scale with surface-layer similarity (Lenderink and Holtslag 2004), the slope of the flux-gradient relations is close to 5 in near-neutral conditions. For , $l_\mathrm{s}$ dominates over the integral length scale.

The modelled mixing efficiency of momentum resembles the formulation proposed by Duynkerke (1991), which was based on measurements from Cabauw. The difference with the Beljaars and Holtslag (1991) formulation, which was also derived from Cabauw observations, demonstrates the increasing uncertainty in the exact formulation of the relations for stronger stratification. Compared to the formulations that are based on observations, the relation used in the ECMWF model clearly applies excessive mixing. In agreement with many observational studies the modelled mixing efficiency of heat is lower than for momentum ($\phi _\mathrm{h} > \phi _\mathrm{m})$. The modelled $\phi _\mathrm{h}$ increases faster with stability than suggested by the formulations proposed by both Duynkerke (1991) and Beljaars and Holtslag (1991), although it is within the range that is observed in other field experiments (Högström 1996).

It is interesting to compare the present results to idealized simulations performed by e.g. van de Wiel et al. (2008) and Ansorge and Mellado (2014). Those studies investigated academic, fully homogeneous and stationary flow, using direct numerical simulations and found strong support for the log-linear (Businger–Dyer) relations. However, it must be realized that turbulent mixing schemes in current realistic models are *also* supposed to represent the effect of, for example, surface heterogeneity (McCabe and Brown 2007), intermittent turbulence (van de Wiel et al. 2002), and gravity waves (Steeneveld et al. 2008). The same processes explain why virtually all observational studies on flux-gradient relations report that for increasing stability the exchange of momentum is far more efficient than the exchange of heat, i.e. $\phi _\mathrm{h} >\phi _\mathrm{m}$ (Beljaars and Holtslag 1991). This justifies additional mixing of momentum compared to results from idealized model studies (Delage 1997; Yagüe et al. 2001).

This observationally-based increase of the mixing efficiency should not be confused with the so-called ‘enhanced mixing’ (i.e. way beyond what is observed in field experiments) that is frequently applied in operational models in order to optimize large-scale model scores (Viterbo et al. 1999).

Details of other components of the SCM can be found in ECMWF (2007). Radiation transport is modelled with the Rapid Radiation Transfer Model (RRTM) scheme. Interactions with the surface and soil dynamics are represented by the Tiled ECMWF Scheme for Surface Exchanges over Land (TESSEL) scheme, which consists of four layers in the soil, a ‘skin layer’ with zero heat capacity, and a vegetation layer. Following Bosveld et al. (2014), we apply a roughness length of 0.15 m for momentum and 0.0015 m for heat.

The SCM simulations are forced with output from daily three-dimensional forecasts of the RACMO 2.1 model (van Meijgaard et al. 2008) starting at 1200 UTC from the ECMWF analysis. The forcing data consist of initial profiles and time-height fields of the geostrophic wind, vertical velocity, and advective tendencies of wind, temperature and humidity.

The SCM simulations are initialized at 1200 UTC and the simulation time is 48 h. Here we use model output with a lead time of between + 24 and + 48 h. No data assimilation or nudging to three-dimensional model fields is applied. The SCM grid consists of 90 vertical levels, and near the surface the grid spacing is roughly 6 m, with the lowest level located at approximately 3 m above the surface. This high-resolution grid configuration is adopted from the GABLS4 intercomparison study (E. Bazile, personal communication, 2017).

## Classification

We classify 11 years of observations and SCM simulations in terms of the horizontal pressure gradient, expressed as the geostrophic wind speed. As the scope of the present study is limited to studying the response of the NBL dynamics to variations in mechanical forcing conditions, nights with overcast conditions were excluded from the analysis. To enable a comparison between summertime and wintertime nights, all nights are synchronized by the moment that the net radiation, $Q_\mathrm{n}$, changes sign from positive to negative. Hereafter, we refer to this moment as $t = 0$ h or ‘the transition’.

To filter out nights with overcast conditions, only nights with an average $Q_\mathrm{n}$ of less than $-\,30\hbox { W }\hbox {m}^{-2}$ were selected. The average is taken over the period $t =$ 0–8 h. To avoid selection of nights with large variations in cloudiness, the standard deviation of $Q_\mathrm{n}$ within this period is required to be less than half the modulus of the average value.

For each night, the ambient $U_\mathrm{g}$ is calculated by taking the average between $t = -\,4\hbox { h}$ and $t=8\hbox { h}$. To guarantee relatively constant values throughout the nights, only nights for which the standard deviation of $U_\mathrm{g} < 1.5\hbox { m }\hbox {s}^{-1}$ are included in the analysis. Selected nights are classified in terms of the average $U_\mathrm{g}$ using a bin-width of $1\hbox { m }\hbox {s}^{-1}$.

The classification is done independently for both the model and the observations. As such, we rather compare nights with similar mechanical forcing conditions, than presenting a one-to-one comparison of observed and modelled nights. As we focus on comparing the observed and modelled response of the NBL dynamics to a changing mechanical forcing this is a legitimate approach. Also, in this way any discrepancies between the dynamical forcings of the model simulations and the actual forcing conditions are avoided. Although the quality of the model forcing data is generally reasonable (bias and root-mean-square error of the modelled $U_\mathrm{g}$ amounts to $-\,0.59$ and $2.21\hbox { m }\hbox {s}^{-1}$, respectively), only 21% of observed and modelled nights are classified in the same bin of $U_\mathrm{g}$. In 10% of the nights the difference is four or more classes of $U_\mathrm{g}$. These differences are a direct consequence of the small bin-width of $1\hbox { m }\hbox {s}^{-1}$ that we apply and the fact that we use model output with a + 24 to + 48 h lead time.

Table 1 indicates the number of nights for each class of $U_\mathrm{g}$ for both the observations and the model. Clearly, although the selected bin-width is very small, for each class of $U_\mathrm{g}$ a significant number of nights is present. Nights with a $U_\mathrm{g}$ value over $16\hbox { m }\hbox {s}^{-1}$ are left out of the analysis due to the small number of nights within these classes.

## Results

Here, the model results are compared to the observations. First, we present the results of the classification in terms of $U_\mathrm{g}$ (Sect. 4.1). Observed and modelled ensemble-averaged time-series of turbulent fluxes and composite profiles of wind speed and potential temperature are shown. This provides valuable insights into the model performance for a gradual increase of the mechanical forcing of the boundary layer. Second, we explore to what extent the model reproduces qualitatively different NBL regimes that have been distinguished in observations (Sect. 4.2). The results are related to recent conceptual findings. Third, the impact of turbulent mixing and the strength of the land-atmosphere coupling are studied by analyzing results of idealized SCM simulations with increasing geostrophic wind speed (Sect. 4.3).

**Table 1:** Number of observed and modelled nights for each class of $U_\mathrm{g}$

$U_\mathrm{g} (\hbox {m } \hbox {s}^{-1}$)	# Obs	# Model
1–2	40	49
2–3	67	98
3–4	84	90
4–5	112	141
5–6	109	133
6–7	115	128
7–8	117	108
8–9	116	121
9–10	103	96
10–11	83	79
11–12	56	60
12–13	54	48
13–14	50	47
14–15	33	35
15–16	28	17

### Classification in Terms of Geostrophic Wind

**Fig. 2:**
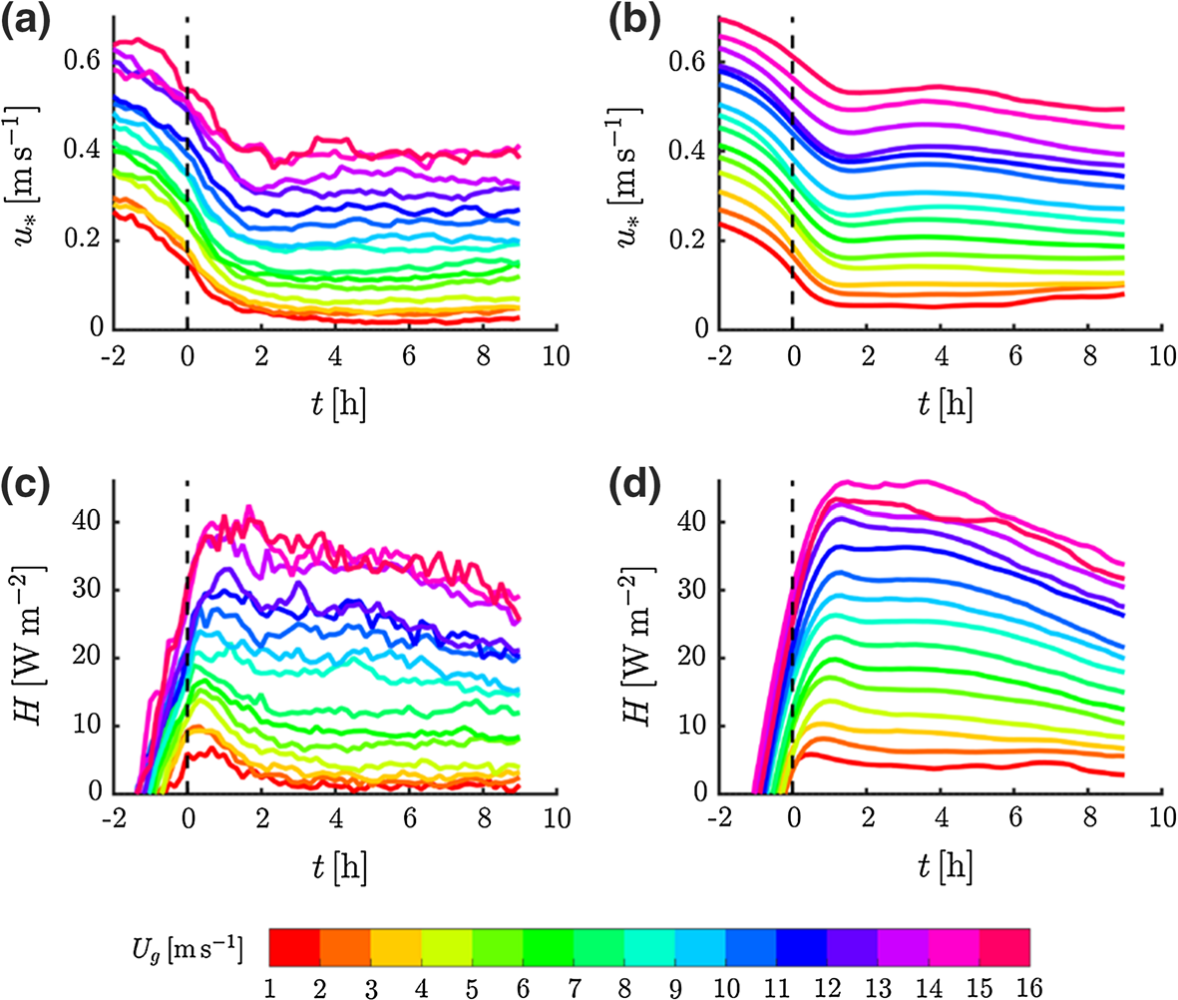
Observed (**a**, **c**) and modelled (**b**, **d**) ensemble-averaged time series of $u_{*}$ (**a**, **b**) and *H* (**c**, **d**) for classes of $U_\mathrm{g}$

Figure 2 shows the ensemble-averaged course of the observed and modelled friction velocity, $u_{*}$, and sensible heat flux, *H*. In general, the model reproduces the temporal evolution of the fluxes and the dependence on $U_\mathrm{g}$ rather well. The magnitude of the fluxes increases monotonically with increasing $U_\mathrm{g}$. Although the modelled turbulent fluxes are somewhat larger than those observed, the general qualitative features appear to be similar to the observations.

For the classes with lower $U_\mathrm{g}$, the ensemble-averaged model results show a much less pronounced maximum in *H* in the first two hours after the transition. On the other hand, inspection of individual days indicates that this feature is present in a substantial part of the modelled nights: for $U_\mathrm{g} \in [2, 3)\hbox { m }\hbox {s}^{-1}$, *H* decreases by more than half between the maximum just after the transition and $t = 4\hbox { h}$ in 44 % of the modelled nights (for the observations this is the case in 84 % of the nights). The observed decrease of *H* over the course of the night (from $t =$ 2–9 h) is reproduced by the model. This trend is related to a similar trend in $Q_\mathrm{n}$ (not shown).

**Fig. 3:**
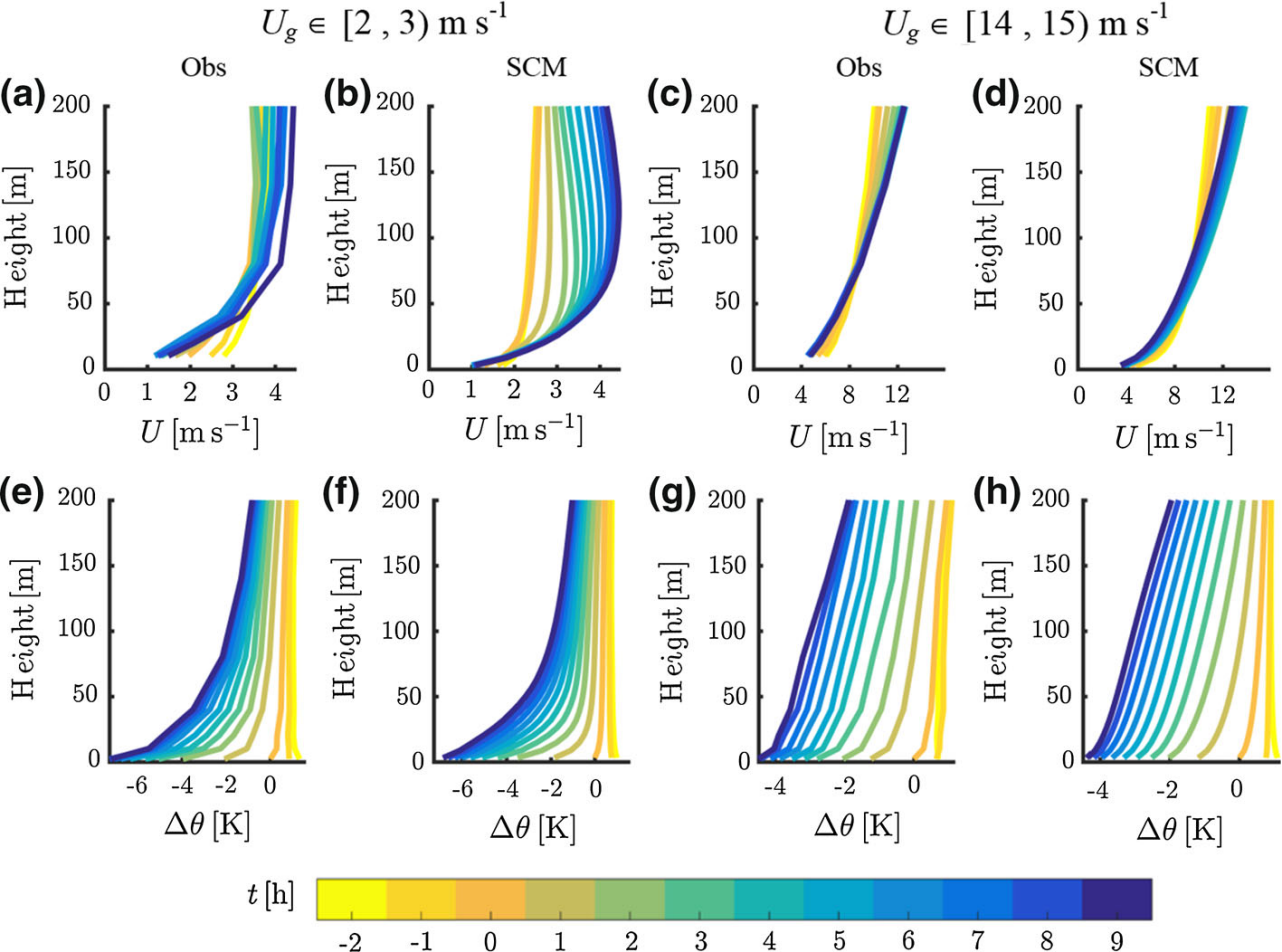
Hourly evolution of observed (Obs) and modelled (SCM) composite profiles of wind speed (top panels) and potential temperature differences (bottom panels) for two classes of $U_\mathrm{g}$ from $t = -2 - 9\hbox { h}$. **a**, **b**, **e**, **f** $U_\mathrm{g} [2, 3)\hbox { m }\hbox {s}^{-1}$. **c**, **d**, **g**, **h** $U_\mathrm{g} \in [14, 15)\hbox { m }\hbox {s}^{-1}$. Potential temperature differences are relative to the 2-m value at $t = 0$. Colours indicate the time relative to the transition

Figure 3 presents composite wind-speed and potential temperature profiles for two selected classes of $U_\mathrm{g}$, representing typically very stable, $U_\mathrm{g} \in [2, 3)\hbox { m }\hbox {s}^{-1}$, and weakly stable conditions, $U_\mathrm{g} \in [14, 15)\hbox { m }\hbox {s}^{-1}$. For both classes, the evolution of the profiles is indicated with hourly intervals from 2 h prior to the transition to 9 h afterwards.

The upper panels of Fig. 3 present the evolution of the composite wind-speed profiles. In very stable conditions, the observations show a decrease of the wind speed below 40 m in the hours prior to the transition. After the transition the wind-speed profile does not change significantly during the entire night. No clear inertial oscillation nor an associated low-level jet occurs as the ageostrophic wind components are small. The modelled profiles are reasonably close to the observed ones from 2 h after the transition to the end of the night. However, during the transition and the preceding late-afternoon, the wind speeds in the model are lower than observed.

In the weakly stable case the wind-speed profile reaches equilibrium shortly after the transition in both the observations and the model results. In the lowest 60 m the wind speed decreases during the hours around the transition, while the upper-tower levels register an increase of the wind speed. As such, a ‘velocity crossing level’ exists at which the wind speed during the night remains relatively constant in time (van de Wiel et al. 2012). Inspection of profiles from other classes of $U_\mathrm{g}$ suggests that the height of this crossing level increases with increasing mechanical forcing (see also van der Linden et al. 2017). This feature is reproduced by the model.

The bottom panels of Fig. 3 present composite profiles of the evolution of potential temperature. The hourly profiles are relative to the 2-m potential temperature at $t = 0$. For both the very stable and the weakly stable case the modelled profiles are close to the observed ones. The shape of the temperature profile depends strongly on the mechanical forcing of the boundary layer (e.g. Estournel and Guedalia 1985; Edwards et al. 2006; Vignon et al. 2017). For the very stable case, the temperature profile has a clear convex shape. Inspection of the modelled profiles of the sensible heat flux indicates that turbulent cooling only occurs in a layer of approximately 40 m thickness (not shown). At higher levels all cooling is the result of radiative flux divergence (Garratt and Brost 1981; McNider et al. 2012). In the weakly stable case the temperature profiles are almost linear. After the initial formation of a shallow surface inversion, a deep turbulent layer cools the NBL in a quasi-steady manner.

**Fig. 4:**
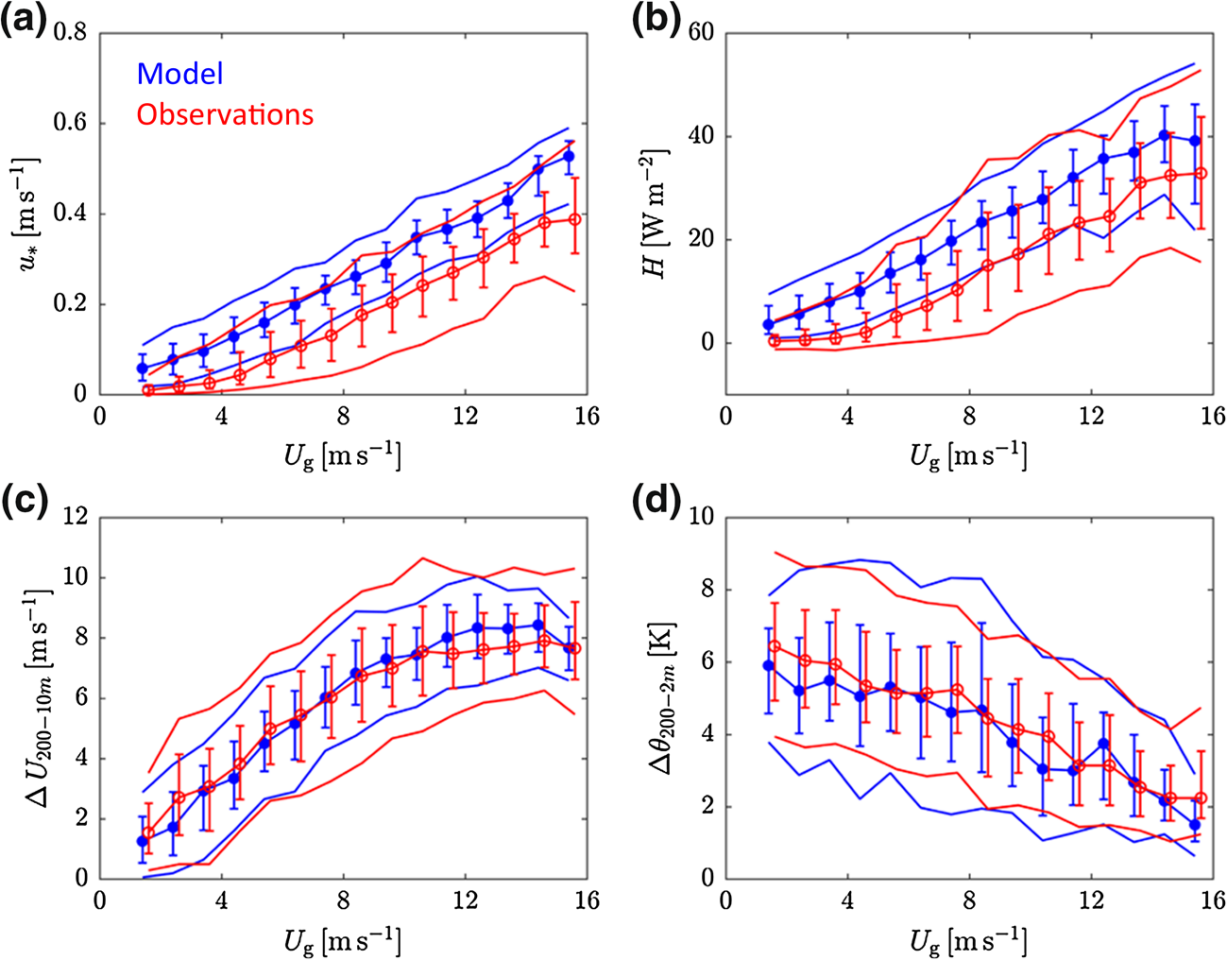
Observed (red) and modelled (blue) distributions of $u_{*}$ (**a**), *H* (**b**), wind shear (**c**), and stratification (**d**) for classes of $U_\mathrm{g}$. Only data between $t = 4$ and $t = 8\hbox { h}$ are included. For each class, the symbol indicates the median value, the error bars the 25th and 75th percentiles, and the outer lines the 10th and 90th percentiles

So far, only ensemble-averaged values of turbulent fluxes (Fig. 2) and vertical profiles (Fig. 3) have been presented. Figure 4 provides more insight in the statistical distribution of the various quantities. Although in general the ensemble-averaged fluxes reveal a gradual ordering (Fig. 2), the data distribution of neighbouring classes overlaps significantly. This is shown in Fig. 4a, b, which presents the distribution of the turbulent fluxes for each class of $$U_\mathrm{g}$$. The percentiles are based on data from the period $$t = $$ 4–8 h after the transition. As can be seen in Fig. 2, in this period the ensemble-averaged fluxes are relatively constant in time. Figure 4a, b shows that the modelled distributions within classes of $$U_\mathrm{g}$$ are reasonably close to the observed ones.

Figure 4c, d presents the distribution of wind shear and stratification (200 m minus near-surface values) for each class of $U_\mathrm{g}$. For both quantities the modelled distributions are very close to the observed ones over the full range of $U_\mathrm{g}$ classes. This may be an unexpected result as the modelled turbulent fluxes are larger than those observed (Fig. 4a, b). However, with a roughness length that depends on wind direction and the presence of internal boundary layers, the observational practice is much more complicated than the relatively simple model environment. As such, no straightforward relation between the magnitude of the surface fluxes and the shape of the vertical profiles exists.

**Fig. 5:**
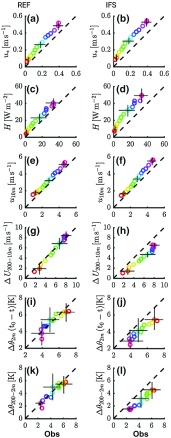
Modelled versus observed ensemble-averaged quantities for the reference model (left panels) and for the IFS mixing scheme (right panels). Data points indicate averages over the period $t =$ 4–8 h. Colours indicate classes of $U_\mathrm{g}$ (colour coding as in Fig. 2). For three contrasting classes of $U_\mathrm{g}$, error bars indicate the 25th and 75th percentile

In Fig. 5, modelled ensemble-averages are compared directly to observed values for different variables. Different colours indicate different classes of $U_\mathrm{g}$. The data points represent average values for the period $t =$ 4–8 h after the transition. For comparison, the SCM simulations were repeated with the IFS mixing scheme (i.e. a first-order turbulent closure scheme with enhanced mixing). The results are included in Fig. 5.

Figure 5a, c confirms that the magnitude of the turbulent fluxes tends to be too large for the reference model. As in Fig. 2, the ensemble-averaged fluxes show a gradual ordering as a function of $U_\mathrm{g}$. However, as indicated by the error-bars, which indicate the 25th and 75th percentiles, the overlap between subsequent classes is significant. The surface fluxes for the IFS mixing scheme are shown in Fig. 5b, d. In this case the overestimation of the surface fluxes is larger than for the reference model.

The reference model slightly overestimates the 10-m wind speed for the whole range of considered geostrophic wind speeds (Fig. 5e). Also the modelled wind shear between 200 and 10 m above the surface is very close to the observed values (Fig. 5g). As a result of the vertical mixing being too intense, the IFS results show a larger overestimation of the 10-m wind speed, in particular for the classes with highest $U_\mathrm{g}$ (Fig. 5f). The wind shear is systematically underestimated (Fig. 5h).

A comparable pattern emerges when considering temperature. Figure 5i, j shows the decrease in $\theta _{2\mathrm{m}}$ since $t = 0$. Although the spread in the data is considerable, the reference model is reasonably close to the observations for the entire mechanical forcing range, while the IFS model underestimates the near-surface cooling. This is consistent with modelled values of the inversion strength, which are close to the observations for the reference model and clearly too low for the IFS model (Fig. 5k, l). The present results agree with many earlier studies reporting that the enhanced mixing applied in most global operational models is detrimental for the representation of the NBL (e.g. Cuxart et al. 2006; Brown et al. 2008; Sandu et al. 2013).

### Nocturnal Boundary-Layer Regimes

Many authors have discussed the occurrence of a maximum in the magnitude of the sensible heat flux for intermediate stratification (e.g. De Bruin 1994; Malhi 1995; Mahrt et al. 1998). This maximum occurs because in near-neutral conditions heat transport through the boundary layer is limited by the very small temperature gradient, whereas in strongly-stratified conditions turbulent transport is limited by the stratification itself. The maximum in the *H*-curve provides a natural separation between a weakly stable (near-neutral) and very stable boundary-layer regime (van Hooijdonk et al. 2015; Monahan et al. 2015).

**Fig. 6:**
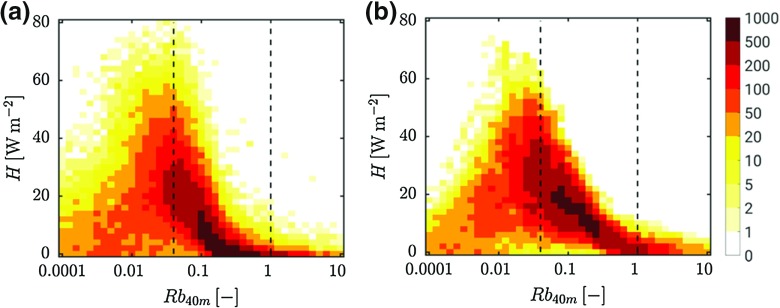
Observed (**a**) and modelled (**b**) relation between *H* and $R_\mathrm{b}$. Colours indicate the number of occurrences. The dashed lines correspond to $R_\mathrm{b} = 0.04$ and $R_\mathrm{b} = 1$

Figure 6 shows the observed (a) and modelled (b) dependence of *H* on the bulk Richardson number, $R_\mathrm{b}$, defined as

2 $$\begin{aligned} R_\mathrm{b} =\frac{g}{\theta }\frac{\Delta z\Delta \theta }{\Delta U^{2}}, \end{aligned}$$

with $\Delta z = 40\hbox { m}$, $\Delta \theta$ is the potential temperature difference between 40 and 2 m and $\Delta U$ is the 40-m wind speed. Figure 6 includes 10-min data from $t = -$ 4 to 9 h for all selected nights. A clear maximum occurs for $R_\mathrm{b} \approx 0.04$ (indicated by a dashed line) for both the observations and the model results. A second line is drawn at $R_\mathrm{b} = 1$ and above this value, *H* is generally very small. In the following, we use those characteristic values of $R_\mathrm{b}$ as a reference. We notice that the exact values have no general validity as they will be different for varying definitions of $R_\mathrm{b}$.

In agreement with the results of Monahan et al. (2015), the data density peaks in the descending branch of the *H*-curve for both the observations and the model results (Fig. 6). At first sight, this may be counter-intuitive as larger gradients lead to smaller turbulent fluxes, leading to even stronger gradients until all turbulence has disappeared (positive feedback). Therefore, it has been argued that this descending branch is dynamically unstable and would therefore occur less frequently in nature. This apparent contradiction has been solved recently by van de Wiel et al. (2017). They showed that in reality negative feedbacks in soil heat transfer and radiation often overrule the aforementioned feedback in the turbulent heat flux. As a result, the formation of stronger and stronger inversions is counteracted. In fact, true instability of the descending branch is only possible over strongly isolating surfaces such as fresh snow or in idealized model simulations where the negative feedback in soil heat transport and radiation is not taken into account. For instance, this occurs when the surface cooling is forced by a fixed heat extraction (e.g. Donda et al. 2015; van Hooijdonk et al. 2017).

**Fig. 7:**
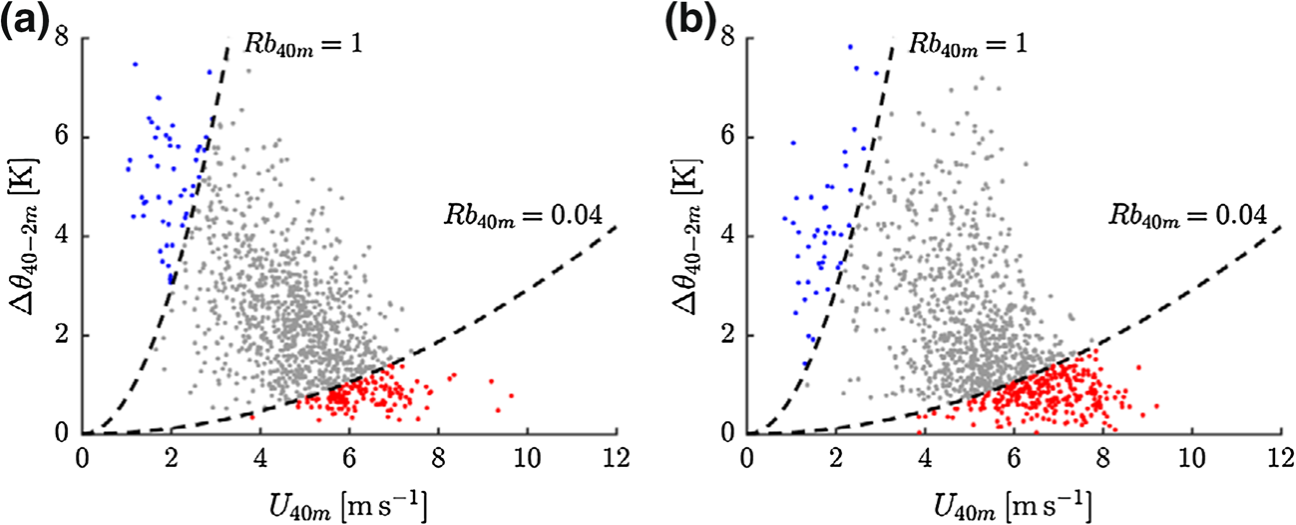
Observed (**a**) and modelled (**b**) relation between near-surface inversion and $U_{40\mathrm{m}}$. Each point represents a single night. Colours indicate the value of $R_\mathrm{b}$ with red $R_{b }<$ 0.04, grey $0.04<R_\mathrm{b} < 1$, and blue $\hbox {R}_{\mathrm{b}} > 1$

Following van de Wiel et al. (2017), we analyze the inversion strength of the lowest 40 m ($\Delta \theta _{40-2\mathrm{m}}$) as a function of the 40-m wind speed, $U_{40\mathrm{m}}$. Each data point in Fig. 7 represents a single night and is based on average values of the inversion strength and $U_{40\mathrm{m}}$ between $t =$ 4–8 h after the transition. Colours indicate the relation with the *H*-curve (Fig. 6), with the red points indicating the ascending branch, the grey points the descending branch, and the blue points vanishing turbulence in strongly stratified conditions.

The overall relation between the inversion and the wind speed appears to be very similar between the observations (a) and the model results (b). For high 40-m wind speeds the inversion stays limited to approximately 1 K. For intermediate wind speeds the inversion strength increases rapidly for decreasing wind speed. As predicted by van de Wiel et al. (2017), the transition between these two regimes coincides with the maximum *H*-curve, indicated by a black-dashed line. The inversion strength is bound to a maximum, which is attained when the downward turbulent transport of warm air is completely suppressed. This occurs for very weak winds.

**Fig. 8:**
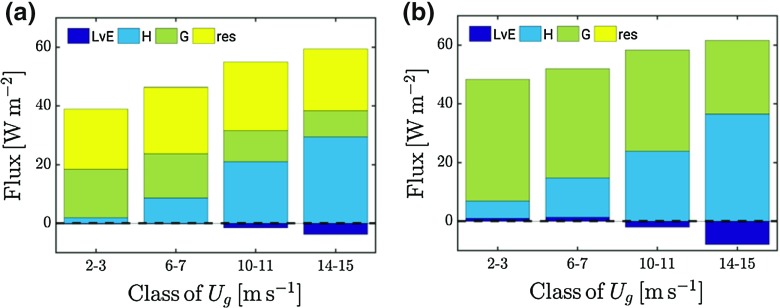
Observed (**a**) and modelled (**b**) components of the surface energy balance for four classes of $U_\mathrm{g}$: [2–3), [6–7), [10–11), [14–15) $\hbox { m}\hbox { s}^{-1}$. The residual term is indicated by ‘res’

Figure 7 indicates that in the absence of turbulence (i.e. the blue data points), the inversion strength is still finite. This can only be achieved if the soil heat flux takes over the role of main heat supplier from the sensible heat flux. This is illustrated in Fig. 8, which shows the contribution of the different terms of the surface energy budget for four classes of $U_\mathrm{g}$. A gradual shift from sensible heat flux to soil heat flux occurs for decreasing $U_\mathrm{g}$ in both the observations and the model results (cf. Sterk et al. 2013). Note that the observed surface energy budget is not closed. A discussion on this well-known problem is outside the scope of the present study, noting that extensive discussions are given in, e.g., Beljaars and Bosveld (1997), de Roode et al. (2010).

It has been argued that ‘enhanced mixing’ is required in order to avoid excessive surface cooling (sometimes called ‘runaway cooling’) as a result of the positive feedback loop between strong stratification and the sensible heat flux (Louis 1979). The present results indicate that to circumvent this problem it appears to be natural to focus on the representation of the soil and the land-atmosphere coupling. We note that very strong surface cooling *can* of course occur in nature over well-isolated (snow-covered) surfaces. For example, Vignon et al. (2017) observed temperature inversions of 25 K over a 10-m height difference at the Dome C observatory at Antarctica.

**Fig. 9:**
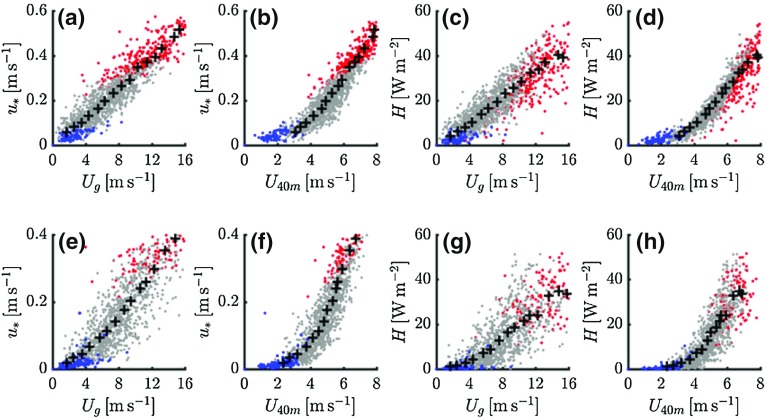
Modelled (top panels) and observed (bottom panels) $u_{*}$ and *H* versus $U_\mathrm{g}$ and $U_{40\mathrm{m}}$. Black pluses indicate ensemble-averaged values for the different classes of $U_\mathrm{g}$. Colours indicate the value of $R_\mathrm{b}$ with red $R_\mathrm{b} < 0.04$, grey $0.04 < R_\mathrm{b} \le 1$, and blue $R_\mathrm{b} > 1$

Recently, several studies explored the occurrence of different NBL regimes by examining the relation between turbulent quantities and the wind speed within the boundary layer (Sun et al. 2012, 2016; van de Wiel et al. 2012; Acevedo et al. 2015; Vignon et al. 2017). In view of these studies, in Fig. 9 we plot $u_{*}$ and *H* versus $U_\mathrm{g}$ (external mechanical forcing) and $U_{40\mathrm{m}}$ (internal parameter) for both the observations and the model results. Each data point represents average values over $t =$ 4–8 h for a single night. Again, the colours indicate the relation to the *H*-curve. (Note that for $u_{*}$ we prefer the local friction velocity (i.e. the eddy-covariance value, see Sect. 2.1) as we now focus on the internal NBL dynamics and not on a direct comparison with model values).

Figure 9a, c indicate that the modelled $u_{*}$ and *H* increase linearly with $U_\mathrm{g}$; the class-averaged values are nearly on a straight line that crosses the origin. There is no sign of a transition between regimes. The observed dependence of $u_{*}$ and *H* on $U_\mathrm{g}$, is not as linear as for the model results (Fig. 9e, g), but still a possible transition between regimes is much more gradual than when the fluxes are related to a wind speed within the boundary layer. The present results resemble those of McNider et al. (2012), who ran their SCM for a broad range of geostrophic wind speeds.

When plotting $u_{*}$ and *H* versus $U_{40\mathrm{m}}$, a clear ‘hockey stick transition’ emerges as reported before by various authors (Sun et al. 2012, 2016; van de Wiel et al. 2012; van Hooijdonk et al. 2015). The relations produced by the model are close to the observed ones, at least in a qualitative way. The ‘kink’ in the hockey sticks coincides with the value of $R_\mathrm{b}$ above which turbulence is weak. The maximum in the *H*-curve occurs for much larger wind speeds.

The question remains why the transition from very stable to weakly stable conditions is much more gradual from the perspective of $U_\mathrm{g}$ than from the perspective of $U_{40\mathrm{m}}$. We suggest that the difference between the two has its origin in a non-linear relation between $U_\mathrm{g}$ and $U_{40\mathrm{m}}$. Model results indicate that for strong stratification the top of the turbulent layer is close to the 40-m level. As a result, in this case $U_{40\mathrm{m}}$ is prone to inertial accelerations. For near-neutral conditions the turbulent layer is much deeper than 40 m. Consequently, $U_{40\mathrm{m}}$ will be substantially reduced by flux divergence.

### Idealized Model Simulations

It has been shown (Fig. 8) that in strongly-stratified conditions the radiative loss at the surface is largely compensated for by the soil heat flux. For these conditions the inversion strength is mainly determined by the strength of the land-atmosphere coupling (Sterk et al. 2013; Bosveld et al. 2014; van de Wiel et al. 2017). Unfortunately, the representation of soil and vegetation processes is complex and involves many uncertain parameters, especially on a spatial scale. Here, we demonstrate the impact of the strength of the land-atmosphere coupling on the modelled inversion strength.

To this end, we defined an idealized model experiment that was run with two different values of the skin-layer conductivity. The reference value used in the model is $10\hbox { W }\hbox { m}^{-2}\hbox { K}^{-1}$, which is a reasonable value for a grassland site such as Cabauw (van den Hurk and Beljaars 1996). As a sensitivity experiment, the reference value was divided by 5. This is probably not realistic for Cabauw, but serves as an illustration for very weak land-atmosphere coupling, for instance in the presence of snow cover. The two permutations were run with both the reference model and the IFS mixing scheme. For each combination of skin conductivity and turbulence scheme, nine simulations were performed with a gradually increasing geostrophic wind speed (0.1, 2, 4, 6, 8, 10, 12, 16, $24\hbox { m }\hbox {s}^{-1}$). Due to the idealized model set-up a direct comparison with the results of the realistic simulations is not straightforward. For instance, the specific humidity was set to very low values to prevent cloud formation leading to increased surface (radiative) cooling rates.

**Fig. 10:**
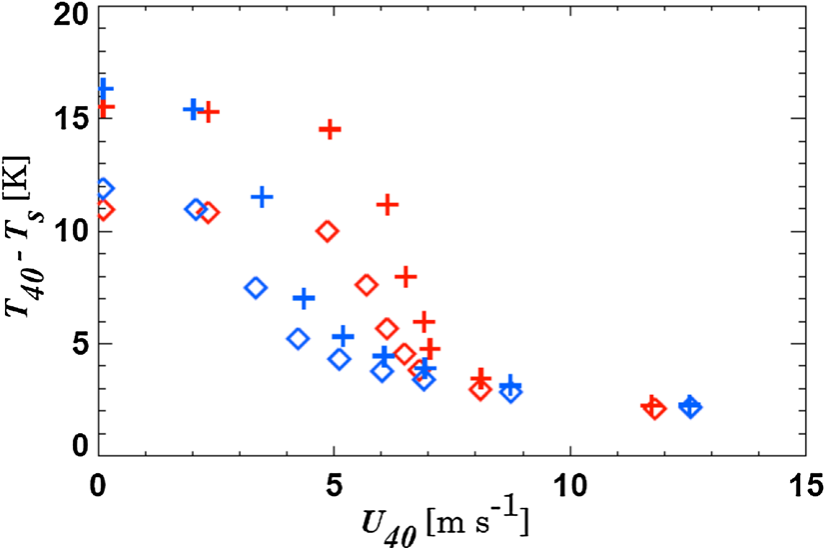
Temperature difference between 40 m and the surface versus $U_{40}$ for idealized SCM simulations. The reference model is indicated in red, the IFS model in blue. Diamonds indicate reference model settings, plus-signs reduced skin-layer conductivity. Each model configuration is run for a suite of nine geostrophic wind speeds

Figure 10 shows the temperature difference between 40 m and the surface as a function of $$U_{40\mathrm{m}}$$. For each of the simulations, average values over the period $$t = $$ 4–8 h are plotted. For large $$U_\mathrm{g}$$, differences between the four sets of simulations are small. For vanishing $$U_\mathrm{g}$$ the inversion strength is almost entirely determined by the strength of the surface coupling and differences between the two vertical-mixing schemes are small. In these conditions turbulence is weak, even in the simulations that apply enhanced mixing. For moderate $$U_\mathrm{g}$$ large differences exist between the two turbulence schemes with weaker inversions and less wind shear in the enhanced mixing simulations.

We note that the temperature difference between 40 and 2 m shows non-linear behaviour for vanishing mechanical forcing: below a certain threshold value the inversion strength increases again for decreasing geostrophic forcing (not shown). This phenomenon that the near-surface temperature increases when the wind speed becomes very small is discussed extensively in McNider et al. (2012) and Sterk et al. (2013).

## Discussion

The present results indicate that NWP models should in principle be able to represent the NBL in a satisfactory way. That is to say, for a wide range of mechanical forcing conditions they should be able to reproduce observed temperature inversions, wind shear, and near-surface parameters with reasonable accuracy (cf. Steeneveld et al. 2006; Sterk et al. 2015).

However, this is in contrast with the general opinion that atmospheric models are not able to represent (strongly) stably-stratified conditions satisfactorily. We explain this as follows. Although turbulence mixing is indeed complex and highly uncertain under strongly-stratified conditions, its absolute contribution to the thermodynamic balance of the boundary layer becomes significantly smaller as the wind speed decreases (Fig. 8). The same holds for the magnitude of the turbulent stresses, which become relatively less important in relation to the other terms in the moment equation. At the same time, we recognize that, given the large vertical gradients in temperature and wind speed, small changes in the model’s mixing properties will have a significant impact on the shape of the profiles and the value of near-surface parameters.

Turbulence parametrizations have often been designed for idealized, homogeneous turbulence, while in model practice they are also supposed to account for patchy, intermittent turbulence. They are even supposed to include the effect of terrain inhomogeneities and/or processes they were never designed for, like (small-scale) gravity-wave drag. The complexity of processes that are responsible for vertical mixing contributes significantly to the modelling problem of the SBL. On the other hand, the fact that leading large-scale models deliberately, albeit for defensible reasons, degenerate the quality of the vertical-mixing parametrization by increasing the mixing efficiency to unrealistic amounts, has in our opinion obscured the discussion of model performance in stably-stratified conditions.

A proper representation of land-atmosphere interactions is key to the realistic modelling of stably-stratified conditions. As demonstrated in the present study, this is particularly true for very stable conditions. Unfortunately, parameters that govern the dynamics of the soil and vegetation are often highly uncertain. Moreover, the spatial variability in land cover, the composition of the soil, and the impact of soil moisture are usually enormous.

Taking into account all the complexities of modelling stably-stratified conditions, the challenge of the present study is relatively straightforward. In an SCM setting, the role of spatial variability is minimal (it is not completely absent as, for example, the actual roughness length depends on the wind direction). As such, it is, for example, not guaranteed that the present settings of the vertical-mixing scheme and the representation of the land-atmosphere interactions will yield equally good results for locations with contrasting surface characteristics or different climates. Still, the current results indicate that the physical parametrizations in large-scale models are in principle sufficiently equipped for modelling stably-stratified conditions for a wide range of forcing conditions.

Finally, we notice that in this study we apply a vertical resolution that is much higher than what is typically used in operational practice (approximately 6 m in the lowest 100 m). Two experiments with coarser grid spacing were performed to study the impact of resolution on the results. The impact of the resolution on the ensemble-averaged wind speed and temperature profiles proved to be small. This is shown in the Appendix, which presents composite temperature profiles for two contrasting classes of $U_\mathrm{g}$. It appears that temperatures at the grid levels are close to each other, while differences in modelled surface temperatures are small. Of course, for individual cases features such as low-level jets and sharp inversions are much better resolved with a fine grid configuration. Although a comprehensive analysis on the impact of vertical resolution would be interesting in itself, it falls beyond the scope of the present study.

## Conclusions

The present work systematically evaluates the performance of an atmospheric single-column model (SCM) for stably-stratified conditions. In particular, the modelled response of the clear-sky nocturnal boundary layer (NBL) dynamics to changing mechanical forcing conditions is investigated using observations from the Cabauw observatory, The Netherlands, as a reference. Therefore, 11 years of model simulations and observations are selected on clear nights and classified in terms of the ambient geostrophic wind speed. The long dataset allows for a small bin-width of only $1\hbox { m }\hbox {s}^{-1}$, which provides a detailed picture of the model’s performance for a broad range of stability conditions, ranging from near-neutral to strongly stratified.

A comparison of modelled and observed ensemble-averaged time series of turbulent fluxes and vertical profiles of wind speed and temperature demonstrates that the model represents the dynamics of the NBL at Cabauw very well for a broad range of mechanical forcing conditions. The model responds realistically to changing mechanical forcing conditions. The model performs well in both near-neutral (weakly stable) and strongly-stratified (very stable) conditions. Observed NBL regime transitions are represented in a natural way. The model resembles characteristics of several conceptual models that study the occurrence of multiple regimes.

The reference model version performs much better than a model version that applies excessive vertical mixing as is applied in several (global) operational models. The detrimental impact of this ‘enhanced mixing’ is obvious for the entire range of considered forcing conditions. The difference between the two model versions illustrates that, on the one hand, NWP models are in principle able to reproduce the NBL for a wide variety of stability conditions but that, on the other hand, in operational practice the interactions between the NBL and other model aspects are still not well understood.

For strongly-stratified conditions, turbulent fluxes constitute only a small fraction of the surface energy budget. In other words, for these conditions the radiative loss at the surface is largely balanced by the soil heat flux. Model sensitivity runs showed that for weak-wind conditions the inversion strength depends much more on details of the land-atmosphere coupling than on the turbulent mixing. The impact of the turbulence scheme is largest for moderate stability.

The presented results indicate that NWP models are in principle able to represent the NBL satisfactorily for a wide range of mechanical forcing conditions. Further research should elucidate whether this conclusion holds for conditions with much weaker land-atmosphere coupling like snow-covered areas or ice-sheets.

## Figures and Tables

**Fig. 11:**
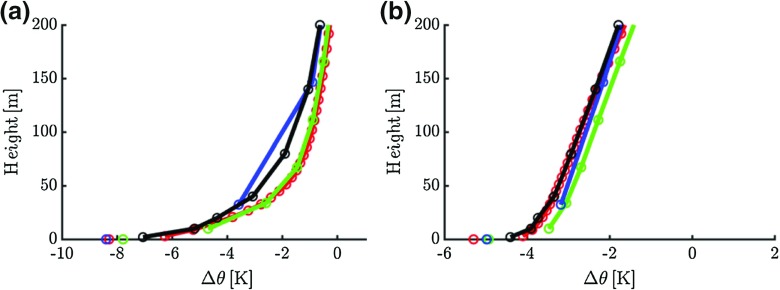
Ensemble-averaged vertical profiles of potential temperature for two classes of $U_\mathrm{g}$: **a** [2,3) **b** [14,15) for the observations (black), the reference model (red), the 91 levels (green) and the 31 levels configuration (blue). Markers indicate model levels. For the three model versions the surface temperature is included as well

## Appendix

To assess the sensitivity of the results to vertical resolution of the SCM, two sets of simulations with reduced resolution were performed. The first had the lowest five grid levels at approximately 10, 34, 67, 111, and 166 m. The second is a very coarse 31-layer configuration with the lowest grid levels at 32, 147, and 350 m. Simulation period (2005–2015), model physics, and classification procedure were equal to those of the reference results. Figure 11 presents ensemble-averaged temperature profiles for two contrasting classes of $U_\mathrm{g}$. From this composite perspective the impact of the enormous differences in resolution are small as the values at the grid levels are close to each other. Also the modelled surface temperatures are remarkably close.
